# Tissue Factor-bearing MPs and the risk of venous thrombosis in cancer patients: A meta-analysis

**DOI:** 10.1038/s41598-018-19889-8

**Published:** 2018-01-26

**Authors:** Chan-juan Cui, Guo-jing Wang, Shuo Yang, Sheng-kai Huang, Rui Qiao, Wei Cui

**Affiliations:** 1 Department of Laboratory Medicine, National Cancer Center/Cancer Hospital, Chinese Academy of Medical Sciences and Peking Union Medical College, Beijing, 100021 China; 2 Department of Laboratory Medicine, Peking University Third Hospital, Haidian District, Beijing, 100191 China

## Abstract

Cancer patients with Tissue Factor (TF)-bearing MPs have been presented association with increased risk of venous thromboembolism (VTE), but results of these studies have not been consistent. We aimed to conduct a meta-analysis to assess the relationship between TF-bearing MPs and risk of VTE in patients with cancer. PubMed, Web of Science and EMBASE Databases were systematically retrieved up to1^th^ June 2017. Two case-control studies and four cohort studies met the entry requirements in this analysis. The summary odd ratio (OR) were estimated by a random effect model. The overall OR was 1.76 (95% CI: 1.21–2.56, I^2^ = 62.0%). The OR of case-control studies was 3.41 (95% CI: 1.45–8.02, I^2^ = 0.0%) and that of cohort studies was1.53 (95% CI: 1.05–2.24, I^2^ = 66.1%). The association between TF-bearing MPs and the risk of VTE in cancer patients was found in this meta-analysis. Publication bias testing and sensitivity subgroup analysis suggested that results of this meta-analysis were robustness. In conclusion, TF-bearing MPs were associated with increased risk of VTE in patients with cancer. Whereas, more well-designed studies and more comprehensive adjustments for confounders in further studies are warranted to affirm the association.

## Introduction

VTE is the most commonly complication in patients with cancer^1^. The rate of VTE is reported in pancreatic cancer 5.3–26%, brain cancer 1.6–26%, gastric cancer 10–15%, lung cancer 1.6–13.6%, and colorectal cancer 3.1–10.2%^2–4^. Although cancer is a known hypercoagulable state with an increased predisposition to VTE, the pathogenesis is rather unclear^5,6^. Tissue factor (TF) has been considered a crucial role in the cancer VTE, especially the expression of TF on microparticles (MPs) which were released from tumor cells in the circulating blood^7–9^. TF is a main initiator of extrinsic coagulation pathway *in vivo*. MPs are small (<1 μm) membrane vesicles, whose phospholipid surface can assemble coagulation factors, resulting in procoagulant potential^8^. The high level of TF-bearing MPs was detected in cancer patients with VTE^9–11^, suggesting that TF- bearing MPs maybe play a role in the development of the cancer VTE.

TF-bearing MPs have been assessed as a high risk factor for cancer-related VTE in several studies^12–15^. Anubha, *et al*.^12^ studied 117 cancer patients including pancreatic, biliary and pancreaticobiliary cancers. The results showed that elevated TF-bearing MPs activity was associated with VTE. The study of Sartori, *et al*.^14^ showed that TF-bearing MPs was also associated with glioblastoma multiforme patients with VTE. However, there have been some studies which show no significant associations were found between elevated TF-bearing MPs and cancer patients with VTE^11,13,16^. Bucciarelli, *et al*.^16^ studied 186 cancer patients compared with controls. The results suggested that there was no association between the high levels of TF-bearing MPs and cancer patients with VTE. Therefore, we conducted the meta-analysis to systematically assess the relationship between TF-bearing MPs and the risk of cancer patients with VTE.

## Methods

### Search strategy

The data were searched up to 1^th^ June 2017 from PubMed, Web of Science and EMBASE Databases. Search terms were used: 1) Microparticles; 2) Tissue Factor; 3) thrombosis OR thromboembolism; 4) cancer OR tumor OR neoplasms. The standard criteria for reporting meta-analyses were followed in this study^17^.

### Study selection

All these articles were screened through the titles and abstracts to find studies that possibly met requirements. Then the full article of eligible studies was read. This analysis included cohort studies and case-control studies that research the relationship between TF-bearing MPs and cancer patients with VTE. These included studies had to meet the following requirements: 1) The outcome was VTE including pulmonary embolism (PE) or deep vein thrombosis (DVT). Thrombosis was diagnosed by standard imaging method. 2) Any type of solid tumor was included in this study. 3) The type of MPs was mainly TF-bearing MPs. 4) Estimates of the relative risk (RR) or odds ratio (OR) or hazard ratio (HR) and its 95% confidence interval (CI) were reported. Review or studies which were impossible to get available information from the published results were excluded.

### Data Extraction

Two independent authors (CC Cui and GG Wang) extracted data from the selected studies. The disagreements were resolved by discussion and consensus. The following data were extracted: the first author’s name, publication year, sample size, cancer type, risk estimates (ORs, RRs and HRs) and 95% confidence intervals.

### Statistical analysis

All of the data were analyzed by STATA, version 12.0 (STATA, College Station, TX, USA) statistical software. We calculated risk estimates (ORs, RRs and HRs) and 95% confidence interval (CI) of developing VTE in cancer patients by TF-bearing MPs. These studies were weighted by the method of inverted variance through STATA software. Heterogeneity was estimated by the I^2^-statistics and Q statistics^18^. P ≤ 0.10 represented statistically significant heterogeneity^19,20^. If På 0.10, then the fixed-effect model was used; Otherwise, the random-effect model was used in this meta-analysis^21,22^ I^2^ is used to measure of how much of this heterogeneity which is due to studies variation rather than sampling error^18^. I^2^-values of 25–50%, 50–75% and >75% represented low, moderate and high heterogeneity, respectively. While a value of I^2^ < 25% represented no significant heterogeneity^18^. Because of the higher potential bias in case-control studies, the subgroup analyses were conducted by the study design of included articles to investigate the sources of possible heterogeneity. We also performed subgroup analyses by the detection method of TF-bearing MPs in included articles. Sensitivity analyses were performed to assess the heterogeneity and robustness of the pooled results.

Potential publication bias was evaluated by the funnel plot (both Begg’s test^23^ and Egger’s test^24^). The quality of these studies was evaluated by the Newcastle-Ottawa Scale (NOS)^25^. Scores of 0–3, 4–6, 7–9 were considered as low, moderate and high quality, respectively.

## Results

### Study selection process and study characteristics

**Figure 1:**
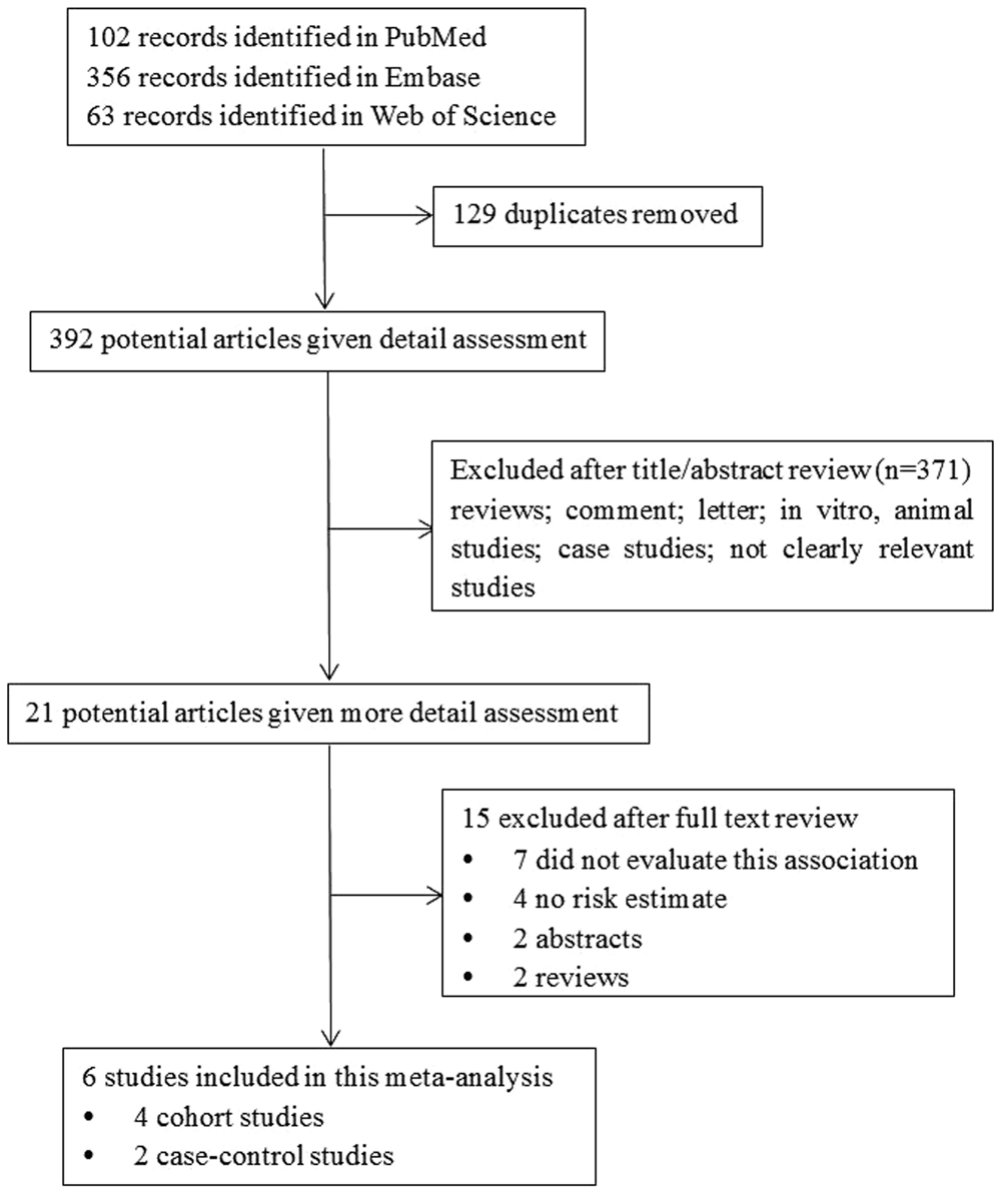
Flow chart of study identification in the meta-analysis.

The flow chart in Fig. 1 showed the process of articles selection. We identified a total of 521 articles (63 from Web of Science, 356 from Embase and 102 from PubMed). Then, 129 duplicates articles were excluded. After reviewing the title and abstract, 371 articles were removed. The remaining 21 articles were given full text review for further evaluation. Finally, 6 articles (4 cohort studies and 2 case-control studies) met the requirements (Table 1). Two of these studies were from USA, three of that were from Europe, and one of that was from Austria (Table 1). The activity assay was used to test TF-bearing MPs in four of these studies. Flow cytometry was used to test TF-bearing MPs in two of these studies (Table 1). In the study of Thaler J., there were three types of cancer (Pancreas, Brain and Colorectal) and the overall HR calculated for VTE were 1.05 (0.76–1.46). The quality of these studies was evaluated by the Newcastle-Ottawa Scale (NOS). Scores of 0–3, 4–6, 7–9 were considered as low, moderate and high quality, respectively^25^. According to the study quality assessment of NOS^25^, one study was scored 8, two studies were scored 7 and three studies were scored 6 (Table 1). So, three of these articles were in high quality while the other three articles were in moderate quality. The adjustments listed in Table 1 were used to adjust the RR/OR/HR in the original studies.

**Table 1:** The characteristics of studies in this meta-analysis. BMI body mass index; MP Microparticle; VTE Venous thromboembolism; PT thromboplastin time; INR international normalized ratio; APTT activited partial thromboplastin time.

**Study**	**Sample size and cancer type**	**Age**	**MPs detection method**	**Follow-up**	**Risk estimates (95%CI)**	**Quality score**	**Adjustments**
Bharthuar A., 2013, USA^12^ Corhort study	Pancreaticobiliary cancers (n = 117)	65 (40–85) years	Activity assay	0.5–1 year	OR 1.40 (1.10–1.60)	6	Age, sex, BMI, race/ethnicity, cell type, d-dimer, number of hospitalizations
Bucciarelli P., 2012, Italy^16^ Corhort study	Cancer patients (n = 186)	45 (11–83) years	Flow cytometry	1 year	OR 2.13 (0.99–4.66)	7	Age, sex, BMI, 95th percentile of MP distribution among controls, factor VIII plasma levels, thrombophilia
Sartori M.T., 2013, Italy^14^ Corhort study	Glioblastoma multiforme (n = 61)	56.7 ± 14.2 years	Flow cytometry	Totally resected cases 445 (281–650) days Subtotally resected cases 227(154–515) days	RR 4.17 (1.57–11.03)	7	PT, INR, fibrinogen, platelet count, APTT, D-dimer, PAI-1 antigen (PAI-1:Ag), and t-PA antigen (t-PA:Ag)
Zwicker J.I., 2009, USA^15^ Case-control study	Cancer with VTE (n = 30) Cancer without VTE (n = 60)	Mean 59.5 years Mean 59 years	Flow cytometry	—	OR 3.72 (1.18–11.76)	6	Age, sex, White blood cell count, Hemoglobin, Platelet Count, Active cancer therapy, Diabetes, Current Smoker
Thaler J., 2012, Austria^13^ Corhort study	Pancreas (n=60) Brain (n=119) Colorectal (n=126)	63 (55–74) 52 (38–64) 63 (55–70)	Activity assay	2 years	HR 1.05 (0.76–1.46)	6	Age, Stage
Campello E., 2011, Italy^11^ Case-control study	Cancer with VTE (n=30) Cancer without VTE (n=30)	Rang 45–89 years Rang 40–92 years	Flow cytometry	—	OR 3.07 (0.54–6.92)	8	Age, sex, platelet count, tumor site, chemotherapy

### Main analysis

**Figure 2:**
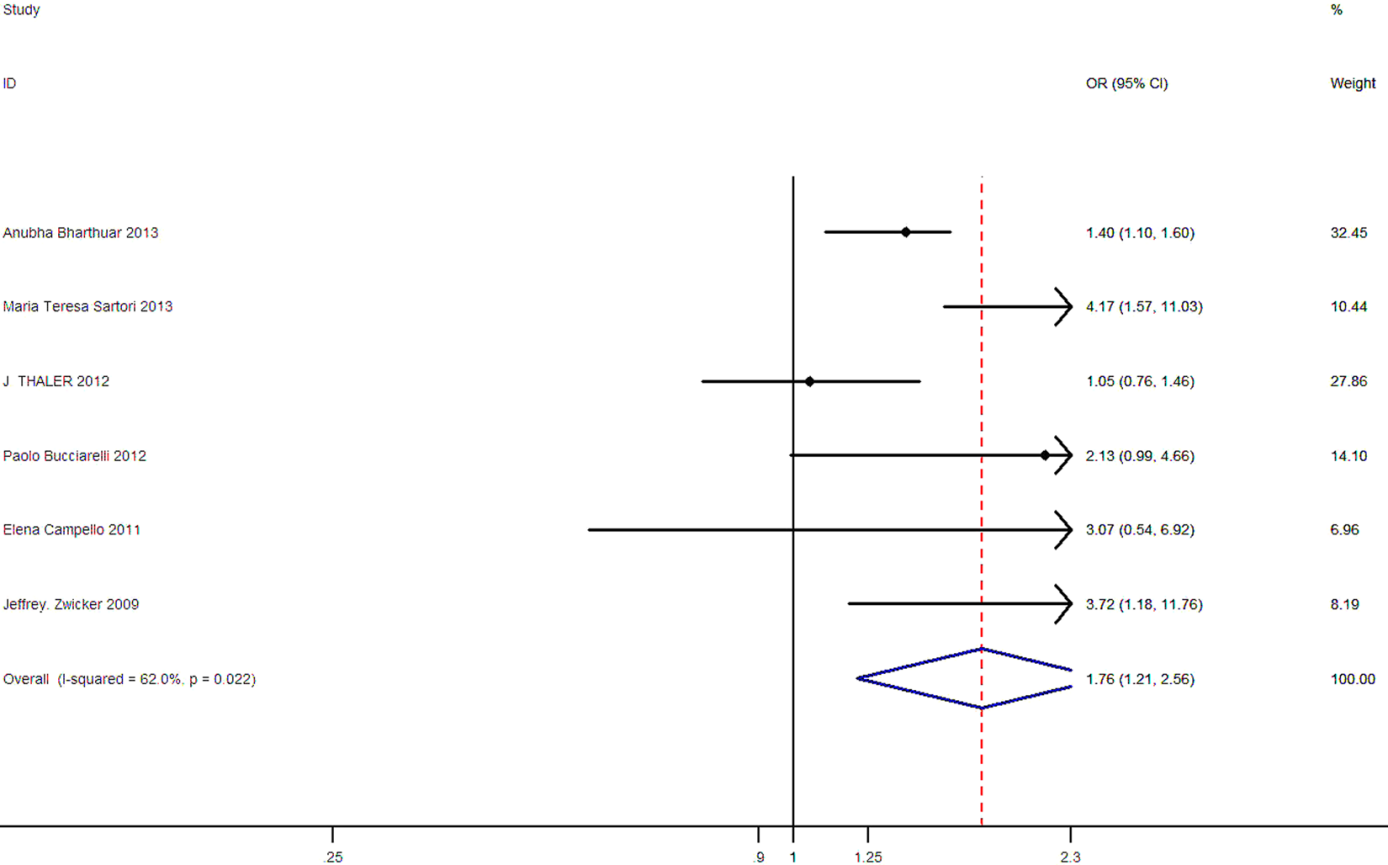
Forest plot of association between TF-bearing MPs and VTE risk in cancer patients, the horizontal lines represent the study-specific OR and 95% CI, respectively. The diamond indicates the pooled results of OR and 95% CI.

In Fig. 2, the forest plot shows the relationship between TF-bearing MPs and the VTE risk in cancer patients. Meta-analysis of these studies presented an increased risk of VTE in patients with high level of TF-bearing MPs compared to those without (Overall OR = 1.76, 95% CI: 1.21–2.56). Moderate heterogeneity was observed in these included studies of this meta-analysis (I^2^ = 62.0%, p = 0.022). Because of the heterogeneity of this meta-analysis was not very good (P ≤ 0.10), then the random-effects model was used in this study.

### Subgroup and sensitivity analysis

**Figure 3:**
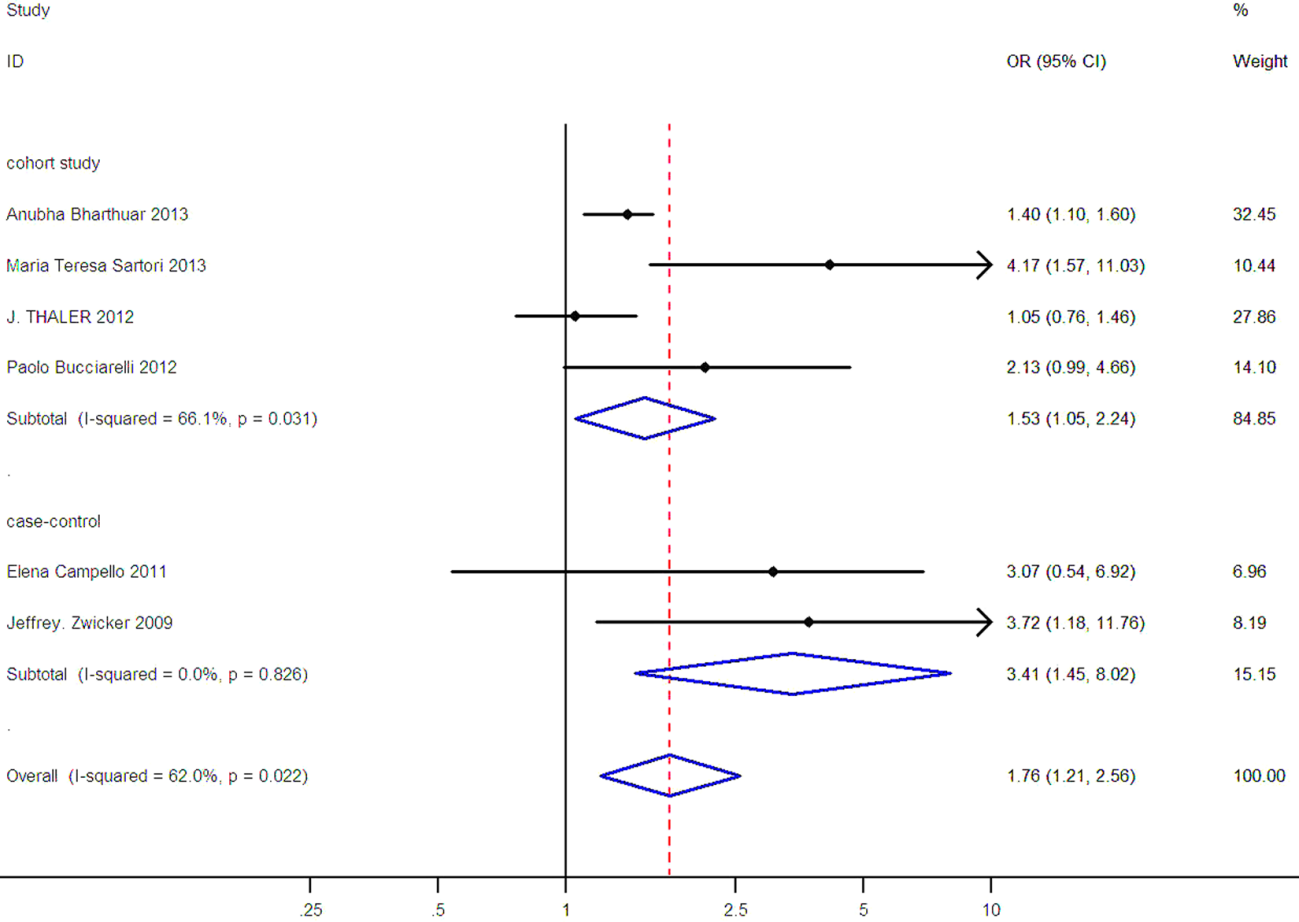
Subgroup analysis of association between TF-bearing MPs and VTE risk by study design, the horizontal lines represent the study-specific OR and 95% CI, respectively. The diamond indicates the summary results of OR with its corresponding 95% CI.

**Figure 4:**
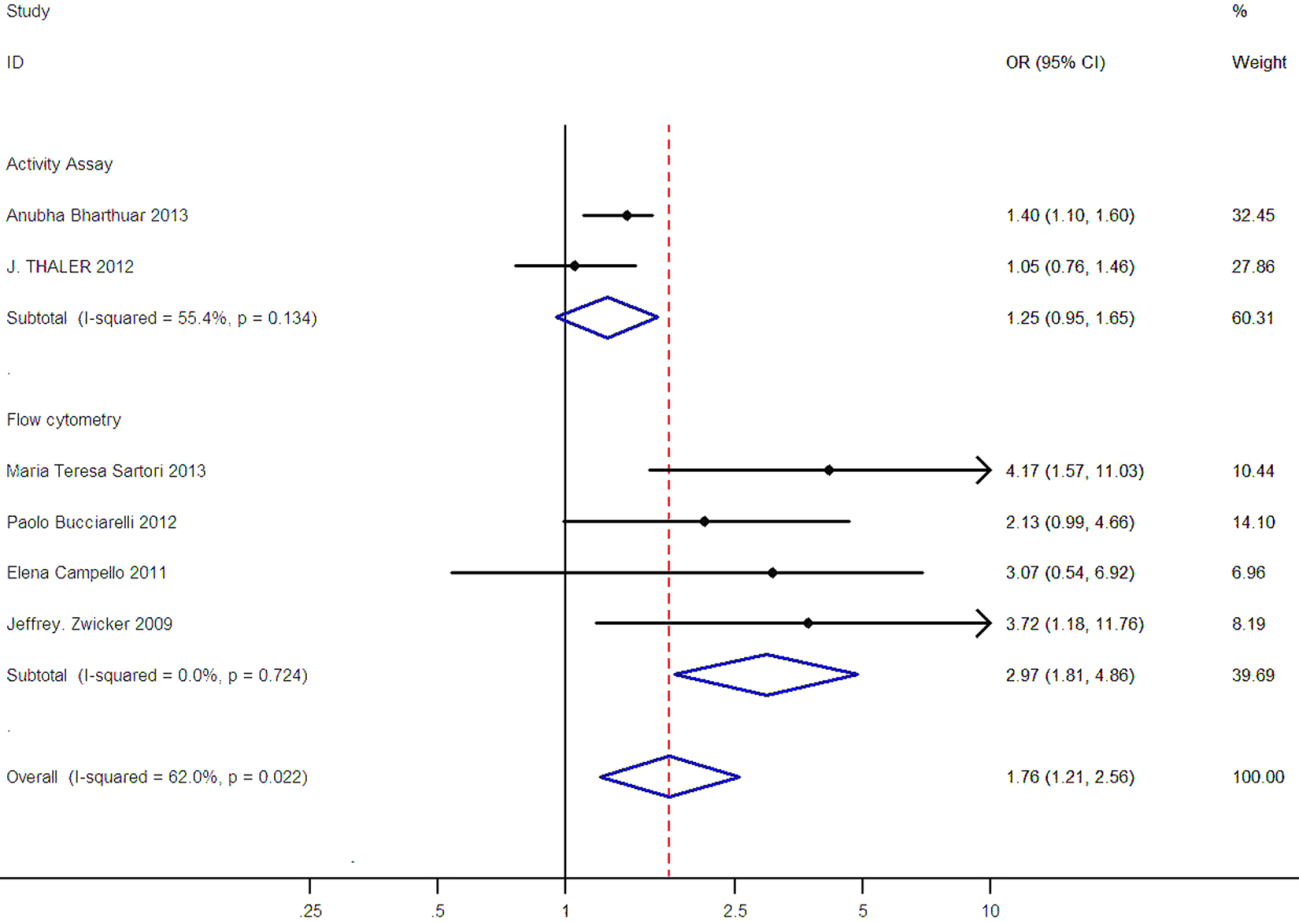
Subgroup analysis of association between TF-bearing MPs and VTE risk by detection method, the horizontal lines represent the study-specific OR and 95% CI, respectively. The diamond indicates the summary results of OR with its corresponding 95% CI.

Subgroup analysis was presented in Figs 3, 4 according to study design and MPs detection method. In Fig. 3, the OR was 3.41 (95% CI: 1.45–8.02, I^2^ = 0.0%) for case-control studies and 1.53 (95% CI: 1.05–2.24, I^2^ = 66.1%) for cohort studies.

In Fig. 4, the OR was 1.25 (95% CI: 0.95–1.65, I^2^ = 55.4%) for activity assay and 2.97 (95% CI: 1.81–4.86, I^2^ = 0.0%) for flow cytometry. For the results using random- model effect model, the positive associations between TF-bearing MPs and VTE risk in cancer patients were similar to the overall result in subgroups.

**Figure 5:**
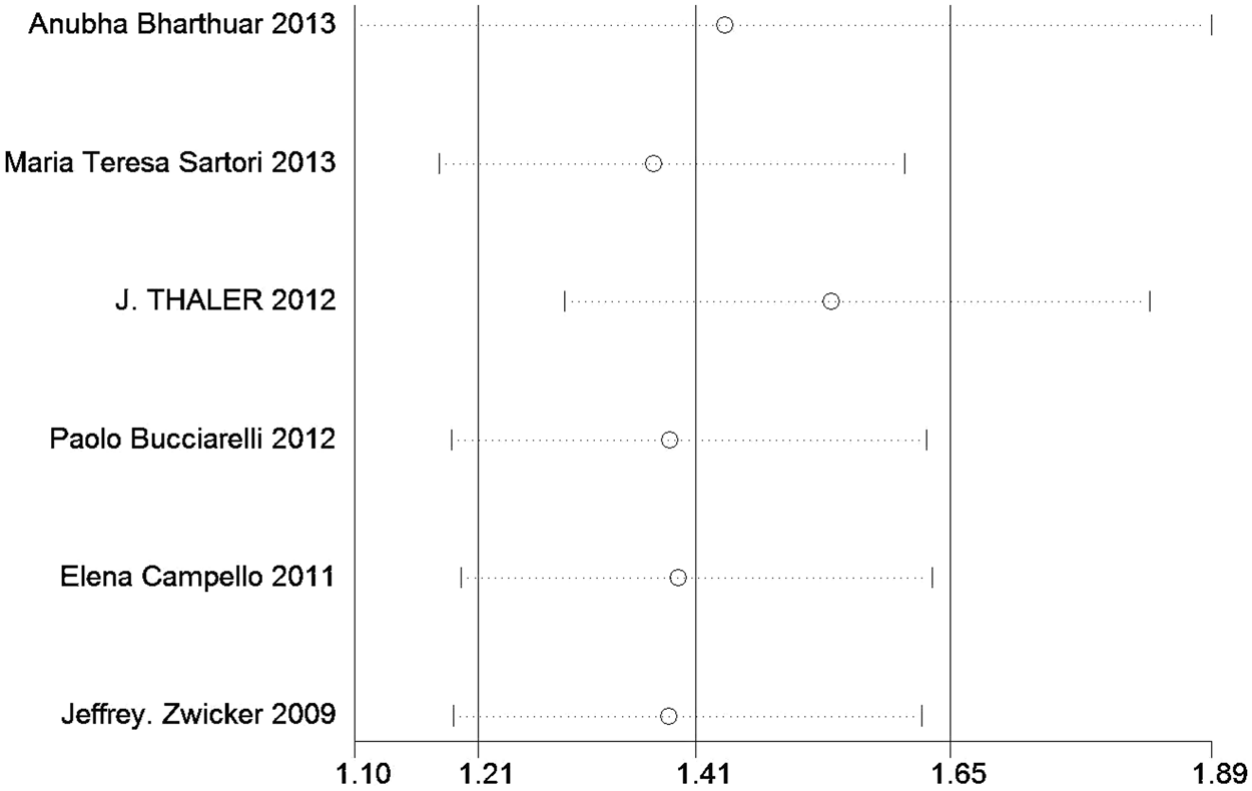
Sensitivity analysis by omitting one study each time, each small circle and dotted lines respectively represent the OR and corresponding 95% CI of the remaining studies after excluding the corresponding research.

Sensitivity analysis was presented by excluded one study at a time and then re-calculating the pooled OR estimates. From Fig. 5, we observed that any of the individual study didn’t influence the pooled OR estimates by fixed or random effects model.

### Publication bias

**Figure 6:**
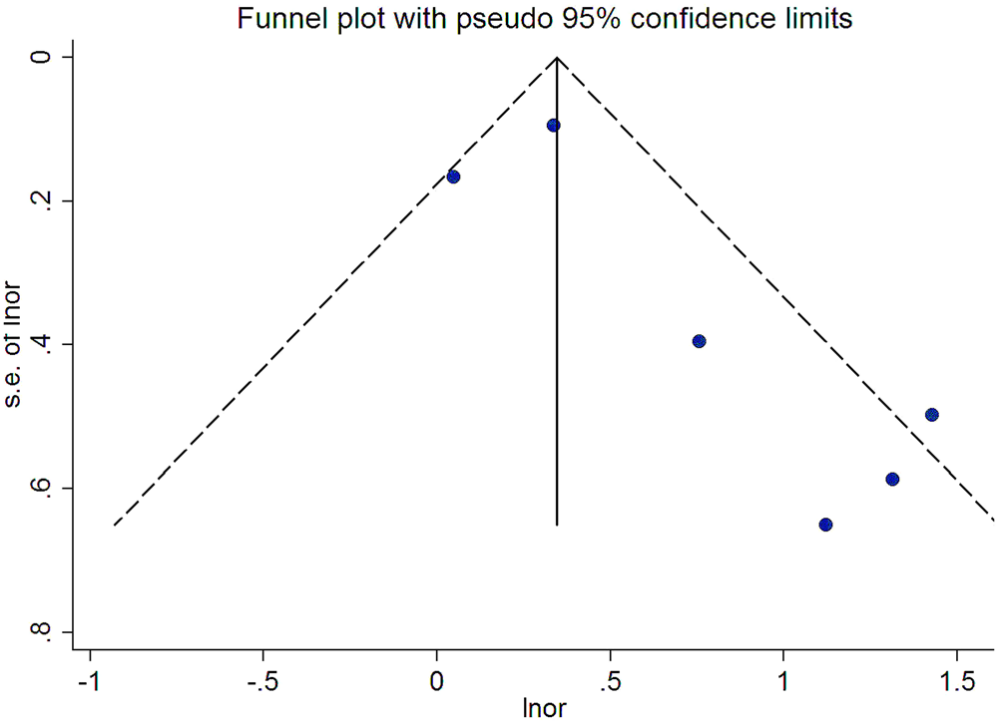
Funnel plot for publication bias test, each point represents a separate study for the indicated association. The horizontal and vertical axis correspond to the OR and confidence limits. s.e., standard error.

The publication bias was evaluated by funnel plots (Fig. 6). There were no publication bias in these studies of the current meta-analysis (Egger’s test P = 0.084 and Begg’s test P = 0.260).

## Discussion

MPs are released as small phospholipid vesicles from the outward blebbing of the plasma membrane after proteolytic cleavage of the cytoskeleton^26^. Some surface proteins are selective packaged into MPs, such as TF and adhesion molecules. MPs are procoagulant largely due to the exposure of negatively charged phospholipids and the presence of TF. The negatively charged phospholipids surface can assemble the positively charged coagulation protein.

To our knowledge, this is the first meta-analysis of the relationship between TF-bearing MPs and cancer patients with VTE. Two independent authors conduct re-sampling data extraction from the selected studies to make sure the robustness of the conclusions^27^. The results of this study show that the TF-bearing MP is an increasing risk factor for cancer patients with VTE. Sensitivity analysis, publication bias testing and subgroup analysis all supported the results in this analysis.

The development of VTE is often observed in cancer especial for its therapy^28–30^. VTE can significantly increase the mortality of cancer patients^31,32^. Therefore, the studies of pathogenesis for cancer VTE gain increasing attention. Cancer cells have been known to express TF on the surface and release TF-bearing MPs^33,34^. A previous study of Hron and colleagues showed that increasing TF-bearing MPs in cancer patients correlated with coagulation activity, as determined by the levels of D-dimer^35^. Subsequently, the retrospective study of Tesselaar and colleagues suggested that the activity of TF-bearing MPs was significantly higher in cancer patients compared with healthy controls and cancer patients with VTE had higher TF-bearing MPs activity than cancer patients without VTE^36^. In this present meta-analysis, an increased risk of VTE in those who had high level of TF-bearing MPs (Overall OR = 1.76, 95% CI: 1.21–2.56, I^2^ = 62.0%, p = 0.022). This result consisted with the studies of Bharthuar, Sartori and Zwicker^8,12,14^.

Our meta-analysis included case-control studies and cohort studies. According to study design subgroup analysis, the case-control studies had higher ORs than the cohort studies. But the case-control studies and cohort studies both showed the TF-bearing MP is an increasing risk factor for VTE in cancer patients. The two results were similar to the overall result. So the case-control studies also provide some value. The OR for case-control studies and cohort studies were 3.41 (95% CI: 1.45–8.02) and 1.53 (95% CI: 1.05–2.24), respectively. The methods of detecting TF-bearing MPs included antigen assays and activity assays^4,37^. The study of Haubold showed that there was no significant correlation between TF-bearing MPs activity assay and antigen assays^38^. But in the study of Geddings, the results presented that there was a strong correlation between TF-bearing MPs activity assay and antigen assay^39^. Therefore, the methods of detecting TF-bearing MPs maybe contribute to the heterogeneity. In our meta-analysis, the subgroup analysis for MPs detection method was conducted. The results showed that OR was 1.25 (95% CI: 0.95–1.65, I^2^ = 55.4%) for activity assay and 2.97 (95% CI: 1.81–4.86, I^2^  =  0.0%) for flow cytometry. The heterogeneity in the subgroups was relatively reduced. Either TF-bearing MPs activity assay or antigen assay suggested that the TF-bearing MP is an increasing risk factor for VTE in cancer patients. There may be some other potential confounding factors that contribute to the heterogeneity, such as sample-sizes, age, cancer treatments. In addition, interpatient and intratumour heterogeneity maybe also have an important role in affecting the result^27^.

In addition to thrombosis, metastasis and recurrence are also the leading cause of cancer related death. There have been many studies of cancer hallmark gene signatures for prognosis^40,41^. However, TF-MPs have been shown to be involved in tumor growth and metastasis. In the study of Zwicker *et al*., 50% of the circulating TF-MPs in patients with pancreatic cancer expressed MUC-1^15^. Similar to the study by Tesselaar *et al*., the plasma levels of TF and MUC-1 double-positive MPs in these patients were reduced after surgical resection of the primary tumor^36^.

There were also limitations in this meta-analysis. First, incidence values for VTE in different types of cancer were different. Brain and pancreatic cancer had the highest rates for VTE at 1.6% to 26% and 5.3% to 26%, respectively^3,4,42,43^. Whereas, prostate and breast cancer had rather low rates for VTE at 0.5% to 1.3% and 0.4% to 8.1%, respectively^3,4,42,43^. Different types of cancer could be analyzed in this study. Cancer heterogeneity maybe affects the result^27^. Because the original studies did not completely separate the types of cancer, it was impossible for us to evaluate the association between TF-bearing MPs and risk of VTE in different types of cancer patients in this meta-analysis. Secondly, case-control studies are more possibly to be interfered by some biases, such as selection bias or recall bias^44^. More factors were considered in cohort studies, so the reliability of cohort studies was higher than case-control studies. Because there were both cohort and case-control studies in our meta-analysis, the recall or selection bias might have affected the estimation of case-control studies and the overall studies. But, in subgroup analysis the case-control studies and cohort studies both showed the TF-bearing MP is an increasing risk factor for VTE in cancer patients, which suggested that selection biases or recall bias did not entirely affect the association. The case-control studies also provide some value. Thirdly, although the publication bias was not observed using the Begg’s and Egger’s test, the two tests had low power to detect the bias in small numbers of studies^23,24^. However, the potential publication bias can’t be totally excluded in this meta-analysis. Because not all potential confounders were adjusted in each study of this analysis, such as cancer treatments, clinical characteristics, cancer types.

In conclusion, this meta-analysis shows that TF-bearing MPs were associated with increased risk of VTE in cancer patients. Whereas, more well-designed studies and more comprehensive adjustments for confounders in further studies are warranted to affirm the association.
